# An Animal Model of Emotional Blunting in Schizophrenia

**DOI:** 10.1371/journal.pone.0001360

**Published:** 2007-12-26

**Authors:** Charmaine Y. Pietersen, Fokko J. Bosker, Janine Doorduin, Minke E. Jongsma, Folkert Postema, Joseph V. Haas, Michael P. Johnson, Tineke Koch, Tony Vladusich, Johan A. den Boer

**Affiliations:** 1 Graduate School of Behavioral and Cognitive Neuroscience, Department of Psychiatry, University Medical Center Groningen, University of Groningen, Groningen, The Netherlands; 2 Eli Lilly and Company, Indianapolis, Indiana, United States of America; James Cook University, Australia

## Abstract

Schizophrenia is often associated with emotional blunting—the diminished ability to respond to emotionally salient stimuli—particularly those stimuli representative of negative emotional states, such as fear. This disturbance may stem from dysfunction of the amygdala, a brain region involved in fear processing. The present article describes a novel animal model of emotional blunting in schizophrenia. This model involves interfering with normal fear processing (classical conditioning) in rats by means of acute ketamine administration. We confirm, in a series of experiments comprised of cFos staining, behavioral analysis and neurochemical determinations, that ketamine interferes with the behavioral expression of fear and with normal fear processing in the amygdala and related brain regions. We further show that the atypical antipsychotic drug clozapine, but not the typical antipsychotic haloperidol nor an experimental glutamate receptor 2/3 agonist, inhibits ketamine's effects and retains normal fear processing in the amygdala at a neurochemical level, despite the observation that fear-related behavior is still inhibited due to ketamine administration. Our results suggest that the relative resistance of emotional blunting to drug treatment may be partially due to an inability of conventional therapies to target the multiple anatomical and functional brain systems involved in emotional processing. A conceptual model reconciling our findings in terms of neurochemistry and behavior is postulated and discussed.

## Introduction

Glutamate and dopamine neurotransmitter systems are distributed throughout the brain and motivate the two main hypotheses underlying the etiology of schizophrenia. The dopamine hypothesis has its origins in the observation that typical antipsychotics (dopamine receptor antagonists) tend to ameliorate positive symptoms [1]–[3]. In comparison, glutamate has been implicated by virtue of the observation that administration of non-competitive NMDA (N-methyl-D-aspartate) receptor antagonists, such as phencyclidine (PCP) and ketamine, to healthy volunteers reproduces not only positive, but also many of the negative symptoms and cognitive impairments seen in schizophrenia [4],[5]. Therefore, in order to address the need for novel antipsychotics that counteract negative symptoms and cognitive deficits associated with chronic schizophrenia, there has been a shift in research from modulating dopaminergic to glutamatergic systems [6].

Negative schizophrenic symptoms include deficits in emotional processing, or emotional blunting, typified by the inability to process fear adequately. In a simple conditioning task using aversive emotional stimuli, for example, patients failed to develop an increase in response frequency to aversively reinforced trials, whereas healthy volunteers acquired a differential response to reinforced versus non-reinforced trials [7] (see also [8],[9]). Many schizophrenic patients manifest deficits in the recognition of fearful faces [10]–[12] in addition to general abnormalities in the processing and attribution of negative emotional states [13],[14].

One brain area that plays a central role in the processing of fear is the amygdala [15],[16]. Importantly, bilateral damage to the amygdala has been shown to impair the processing of fearful facial expressions in otherwise healthy human subjects [17] and reduced amygdala volumes have been found in schizophrenic patients [18]–[21]. In fact, some neuro-imaging studies suggest that the positive symptoms of schizophrenia are associated with increased amygdala activity, whereas negative symptoms are associated with hypoactivation [22],[23].

In line with the above observations, Aleman and Kahn [24] propose a two-hit model of amygdala abnormalities in schizophrenia, combining the glutamate neurotransmitter hypothesis with amygdala dysfunction. They speculate that prolonged activation of the amygdala during psychotic states (positive symptoms) in the onset stages of schizophrenia could lead to glutamate excitotoxicity, resulting in amygdala lesions and long-term hypofunctioning (see also [25],[6]), which ultimately could underlie the negative symptoms of the disorder.

In order to investigate deficits in emotional processing, we examine fear conditioning in the rat—an animal that has provided the basis for several extant models of schizophrenia [26]–[30]. Specifically, we attempt to simulate the putative amygdala hypoactivation caused by glutamate excitotoxicity indirectly by blocking the NMDA receptor. We achieve this by administering the glutamate NMDA-receptor antagonist, ketamine, to rats prior to fear conditioning. This model for the etiology of negative symptoms is consistent with many previous studies showing that the NMDA receptor is involved in fear conditioning. Goosens and Maren [31], for example, have shown that infusion of the NMDA antagonist D, L-2-amino-5-phosphonovalerate (APV) into either the basolateral or central nuclei of the amygdala blocks the acquisition of conditional fear. We hypothesize that the hypoglutamatergic state induced by ketamine administration will interfere with normal fear processing due to abnormalities in basic association of fear cues in the amygdala and related brain areas (see also [32],[33]).

To measure the effects of these manipulations, we examine a behavioral assay of fear conditioning known as freezing (i.e. absence of movement in response to conditioning), either in the presence or absence of administered ketamine. We also measure two neural assays of fear conditioning within two separate regions of the amygdala (the central [CEA] and basolateral nuclei [BLA]). One assay is cFos expression—a measure of learning-related neural activity [34]. The second assay is neurotransmitter tissue content, which indicates the amount of neurotransmitter (intra- and extracellular) in a given brain region.

In addition to the amygdala, we also examine various brain areas associated with emotional processing, including the anterior cingulate cortex (ACC) and the nucleus accumbens (core and shell, Nacc). The rat ACC, a sub-area of the prefrontal cortex, has previously been shown to be involved in associative learning, particularly fear conditioning [35],[36], and in cognitive processes, such as attention [37],[38]. Lesions of this area in humans produce symptoms including apathy, inattention, dysregulation of autonomic function and emotional instability [39], all symptoms present in schizophrenic patients. The Nacc has also been implicated in the neurobiology of schizophrenia [27] and is an area primarily involved in motivation [40],[41]. It is also intimately linked with the ACC [27],[37] and BLA [42] and receives glutamatergic projections from these areas.

It has previously been reported that ketamine administration, in addition to affecting the glutamate system, also affects the dopamine system [43],[44]. Dopamine D_2_ receptor antagonists have also been found to ameliorate ketamine-induced impairment of some prefrontal cortex-dependent cognitive functions in rodents [45]. A single-neurotransmitter perturbation is therefore probably not sufficient to fully describe the emotional deficits reflected in the schizophrenic brain. Therefore, both neurotransmitter systems implicated in the origins of schizophrenia, as well as their interactions, are investigated here (i.e. through measurement of neurotransmitter tissue content).

In order to validate the etiological aspects of our model, we administer two antipsychotics used in the clinical setting, haloperidol and clozapine. Haloperidol, a typical antipsychotic, is used for treating positive symptoms of schizophrenia [1],[46],[2] [3]. Clozapine, in contrast, is an atypical antipsychotic that has been found to alleviate negative and cognitive symptoms of schizophrenia [6]. Clozapine also differs from conventional neuroleptics, such as haloperidol, in the way it affects the glutamate system [47],[6]. For example, animal studies have shown an increase in medial prefrontal cortical glutamate concentrations after clozapine administration, while haloperidol did not elicit this increase [48]. Another animal study, comparing the effects of haloperidol and clozapine on ketamine-induced alterations in metabolism, found that clozapine completely blocked the effects of ketamine in several brain areas, whereas haloperidol did not [49]. We therefore hypothesize that clozapine, but not haloperidol, will retain the behavioral changes induced by fear conditioning following ketamine administration. We also administer a new compound (LY 379268; (-)-2-Oxa-4-aminobicyclo [3.1.0.] hexane-4,6-dicarboxylate), a metabotropic glutamate 2/3-receptor agonist, which is currently being tested for its involvement in fear learning [50]. It is presently unclear whether LY 379268 can affect conditional fear processing in the rat. A recent study, however, does suggest that agonists of this receptor possess anxiolytic properties [51]. The metabotropic glutamate 2/3-receptor is located primarily in forebrain regions, and LY 379268 has been shown to decrease glutamate release in these areas [52]. We therefore postulate that LY 379268, as a glutamate agonist, will inhibit ketamine's actions on glutamate content, especially in forebrain areas, in line with similar studies in the literature [53]–[55].

To summarize, we hypothesize that the influence of ketamine on fear conditioning will manifest itself as a decrease in neuronal activity, relative to fear-conditioned saline controls, in brain regions associated with fear processing, in addition to inhibiting behaviors typically derived from fear conditioning. We also hypothesize that ketamine will interfere with the neurochemical alterations in brain areas associated with fear conditioning, primarily through action on NMDA receptors in the amygdala. Further, we expect that clozapine will preserve normal fear-conditioned behavior and neuronal activity abolished by ketamine. We also hypothesize that haloperidol, as it mainly affects positive symptoms, will not inhibit ketamine's actions in these assays. We also tentatively postulate that LY 379268 will prevent ketamine's actions assessed in the above-mentioned assays, mainly in forebrain areas. Evidence in favor of these hypotheses would support the notion that glutamatergic hypofunctioning in the amygdala and related brain areas underlies negative schizophrenic symptoms, thereby paving the way for future studies to explore novel drug treatments of these notoriously drug-resistant symptoms.

## Materials and methods

Two experiments were conducted to measure the alterations in behavior, cFos expression and neurotransmitter content due to fear conditioning and ketamine and/or antipsychotic administration. This was done because the same animals could not be used for both experiments due to methodological constraints.

### Animal Housing

All animals were cared for in accordance with the principles laid down by the European Communities Council Directive (1986) for the Protection of Vertebrate Animals used for Experimental or Other Scientific Purposes (86/EEC), which is comparable to the guidelines laid down in the “Principles of laboratory and animal care.” Male Sprague-Dawley rats weighing between 225–250 g were obtained from the central animal facility (Groningen, The Netherlands) and were housed individually in a temperature (±23°C) and humidity controlled (40 to 60%) environment. Food and water were delivered *ad libitum*.

### Drugs

Haloperidol (0.25 mg/kg, i.p.) was diluted from 5 mg/1ml Haldol® injection capsules. Both clozapine (5 mg/kg, i.p.) and ketamine (16 mg/kg, s.c.) were dissolved in physiological saline (0.9%), with hydrochloric acid (HCL) added to clozapine to aid dissolving. One µl/ml of 5N sodium hydroxide (NaOH) was added to the LY 379268 (3 mg/kg, s.c.) in saline solution before sonication, also for dissolving purposes. Antipsychotics were administered 30 minutes prior to ketamine, in accordance with previous studies investigating the effects of antipsychotics on NMDA antagonists [56]–[58]. All drug doses were determined empirically, i.e. it was the highest dose possible that did not affect locomotor behavior or induce catalepsy, except ketamine (see below). The clozapine and haloperidol doses are in line with the clinical setting, as determined by D_2_ receptor occupancy [59]. Clozapine was obtained from Sandoz Pharma AG, Switzerland; haloperidol from Janssen-Cilag, The Netherlands; LY 379268 from Eli Lilly, USA; and ketamine hydrochloride from Sigma, Germany.

### Shock Construct

**Figure 1:**
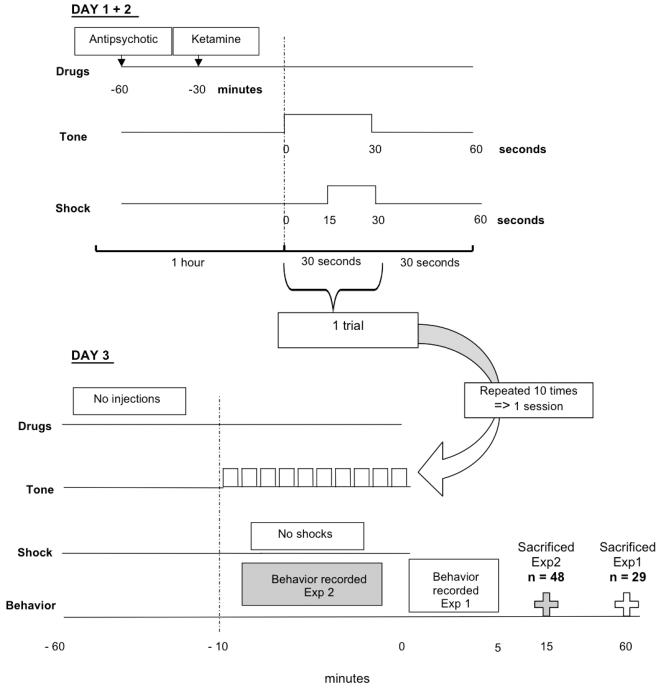
Injection and shock schedule. One trial consisted of a 30 second period. During the 30 seconds, a tone was emitted. Within the second half (15 seconds) of this 30-second period, the shock was delivered. Thirty seconds following the trial served as a rest period. All trials took place in the morning and were repeated consecutively ten times per day, resulting in one session lasting 10 minutes in total. Control rats followed the same routine with tone emission, but without experiencing any shocks. On the third day, the same procedure was followed, but without administering shocks. This was done to avoid measuring behavioral outputs due to direct drug interference or pain stimuli. *Experiment 1: n = 48; experiment 2: n = 29*

The shock box was a specially constructed wooden container with a floor made of a metal grid. A central computer controlled the current and tone emission making use of a program that was specially developed for this study (N594 version 2.00, University of Groningen, The Netherlands, 2002). All shock trials took place in the mornings. Rats destined to undergo fear conditioning were subjected to a shock (1.5 mA) that was paired with a tone (60 dB tone) during conditioning trials (Fig. 1). This shock intensity was based on a pilot study indicating that 1.0 and 1.5 mA shocks induced comparable stress levels (corticosterone and behavior), but that the latter shock intensity was superior in terms of variability of all incurred stress parameters [60].

### Fear conditioning paradigm

After arrival from the animal breeding facility, rats were allowed to acclimatize for two to three days. They were then handled daily for five days in order to eliminate handling stress as a confounding variable. All drug injections only took place on the first two days of the three-day conditioning paradigm, i.e. only during the actual conditioning phase of the experiment. Injections were omitted on the third day of conditioning testing to avoid unnecessary drug interaction with behavioral measurements. Specifically, haloperidol, clozapine and LY 379268 were administered half an hour before ketamine injections in experiment 1 (see Fig. 1). Only clozapine was used in experiment 2 and was injected at the same time point as in experiment 1. Ketamine injections and saline shams were administered half an hour before fear conditioning, as previous observations in our lab showed that half an hour was sufficient for ketamine-induced increases in locomotor activity to subside [61].

The rats were subsequently placed individually in the shock box. One trial consisted of a 1-minute period. We presented rats with a tone during the first 15 seconds. In the next 15 seconds, the tone was emitted in combination with a shock. Thirty seconds thereafter, the process was repeated. This 1-minute trial was repeated 10 times per day in succession, resulting in one session of 10 minutes (or 10 trials). This protocol was repeated on day 2. Control rats followed the same routine with tone emission, but without experiencing any shocks, essentially following a timed tone protocol. On day 3, neither group received shocks or injections; otherwise the animals follow the same protocol. This was done to avoid measuring behavioral outputs due to direct drug interference or pain stimuli.

Following this fear conditioning paradigm, behavior was recorded (Philips Explorer Camcorder) for 5 minutes after the test session (experiment 1) on the third day in order to determine if a fear response was acquired in reaction to the whole stress procedure (tone and context). Previous studies in our lab (unpublished data) have shown that minimal extinction occurs during the first 5 minutes after the last test session and that fear-conditioned freezing behavior was still evident. In order to adequately estimate the immediate effects of altered neurotransmitter content (which degrades quickly) on behavior, freezing was measured during, rather than after, the test session in experiment 2 and animals sacrificed 15 minutes thereafter.

### Behavioral measurements

Behaviors were subsequently analyzed with The Observer (Noldus version 3.0, The Netherlands). An independent observer, unaware of experimental conditions, noted both the frequency and total duration of freezing (experiments 1 and 2), grooming, rearing and resting behavior (experiment 1). Freezing was denoted as an absence of any movement (not sleeping), except for respiration and whisker twitching. Rearing was defined as the raising of the body onto the hind legs, while resting served as a default state when none of the other behaviors were being displayed (i.e. normal ambulatory behavior). Freezing behavior, as well as being a behavioral expression of stress, is also the main assay of fear conditioning [62],[63]. As this parameter gave the best results in experiment 1, we eliminated all other behavioral measurements from the design in experiment 2.

### Design of Experiment 1

**Figure 2:**
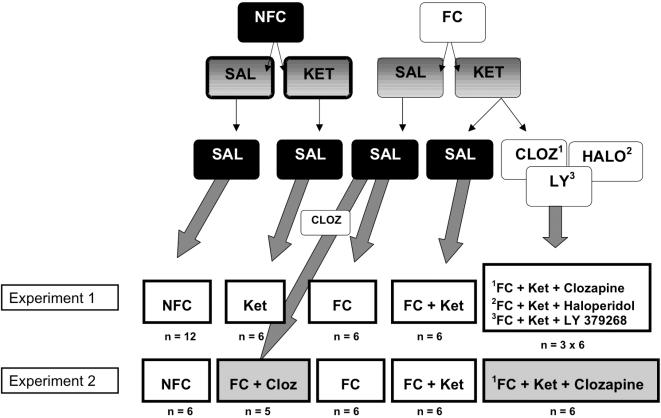
Experimental group divisions. Diagram portraying the rat group divisions. At the top of the hierarchy, we divided rats into two main groups: those receiving fear conditioning, and those not. Those animals receiving fear conditioning, were then further divided into rats receiving ketamine administration and rats receiving saline shams. The latter group would form the fear conditioning only group (FC). The rats not receiving fear conditioning were also divided into two groups depending on whether they would receive a ketamine or saline injection; the former group making up the ketamine only group (Ket), and the latter being the control group (NFC). The remaining fear conditioned rats also receiving ketamine were then further divided into those receiving either a saline injection (FC+Ket) or those receiving an additional antipsychotic injection consisting of clozapine (FC+Ket+CLOZ), haloperidol (FC+Ket+HALO), or LY 379268 (FC+Ket+LY). *CLOZ, clozapine; FC, Fear conditioning; HALO, haloperidol; KET, ketamine; LY, LY379268; NFC, no fear conditioning; SAL, saline.*

The rats (n = 48) were divided into seven groups as illustrated in Fig. 2. At the top of the hierarchy, we had a fear conditioned (FC, n = 6) group, a fear conditioned with ketamine administration (FC+Ket, n = 6) group, a non-fear-conditioned (NFC, n = 12) group, and a non-fear-conditioned group that received ketamine (n = 6). An additional four groups receiving the FC+Ket treatment also received antipsychotics; each group received either a clozapine (FC+Ket+CLOZ; n = 6), haloperidol (FC+Ket+HALO; n = 6), or LY 379268 (FC+Ket+LY; n = 6) injection, in addition to ketamine and fear conditioning.

### cFos expression: perfusion and preparation

One hour after the end of the test session (day 3), the rats were perfused trans-cardially with 4% paraformaldehyde (Merck, Germany) for 20 minutes. This time point was chosen so as to incorporate all events happening in the brain during the tone signals in the testing session on day 3 [64]. The brains were then removed and placed into 4% paraformaldehyde, and kept at 6°C for two days. Thereafter, they were transferred into 0.02 M potassium phosphate buffered saline (PBS; pH 7.4) with 1% sodium azide (Boom, Meppel, The Netherlands) to prevent bacterial growth and were stored at 6°C. In preparation for cFos staining, whole brains were dehydrated in a 30% sucrose solution overnight and subsequently frozen with gaseous CO_2_ at −80°C. The brains were cut using the Leica CM 3050 cryostat machine at 40 micrometers thin slices and stored at 6°C in 0.02 M PBS buffer (pH 7.4).

### cFos staining: Immunocytochemistry

Coronal cryostat sections of 40 mm were collected in 0.01 M Tris buffered saline (TBS, pH 7.4) and rinsed 3 times, 5 minutes per rinse (3×5 min). After pre-incubation with 0.3% H_2_O_2_ (10 min, in 0.01 M TBS, pH 7.4), the sections were washed with 0.01 M TBS (4×5 min, pH 7.4) and incubated with a rabbit polyclonal antibody raised against cFos (Ab-5 Oncogene Research Products, Calbiochem, 1:10.000 in 0.01 M TBS-Triton 0.01%, 4% normal goat serum) for 48–60 hours at room temperature. Subsequently, the sections were washed in 0.01 M TBS (8×5 min, pH 7.4) and incubated for 2 hours at room temperature with biotinylated goat anti-Rabbit IgG (Vector, 1:1000 in 0.01 M TBS). After rinsing with 0.01 M TBS (6×5 min, pH 7.4), the immunoreactivity was visualized with a standard ABC method (Vectastain ABC kit, Vector, (1 drop A+1 drop B)/20 ml TBS for 2 hours). After washing with TBS 0.01 M (6×5 min, pH 7.4) the peroxidase reaction was developed with a di-aminobenzidine (DAB)-nickel solution and 0.3% H_2_O_2_ (0.5 mg DAB/ml Distilled water; 1.0% nickel ammonium sulphate (NAS)) in 0.1 M sodium acetate (NaAc, pH 6.0). To stop the reaction, the sections were washed with 0.1M NaAc, pH 6.0 (3×5minutes) and then 0.01 M TBS (3×5 min, pH 7.4) and were subsequently mounted on gelatin-coated slides, air dried, dehydrated, and coverslipped with DePeX (Gurr) (Boom, Meppel, The Netherlands).

The area of the region of interest was measured and, after background correction, the number of immunopositive nuclei was quantified using a computerized image analysis system (Leica Qwin version 2.3, Leica Microsystems Imaging Solutions). The average number of cFos immunoreactive cells was calculated and expressed as number of positive nuclei or Counts/Area (0.1 mm^2^). Areas included in the cFos analysis were: the paraventricular nucleus (PVN), CEA, BLA (subdivided into anterior and posterior nuclei) and lateral nucleus of the amygdala, Nacc (core and shell), and ACC. The Swanson [65] co-ordinates (rostral-caudal) are given in Table 1 as millimeters from Bregma.

**Table 1:** Brain areas: Swanson (1992) rostral-caudal stereotaxic co-ordinates

EXPERIMENT 1	
Area	mm from Bregma
Anterior cingulate	+2.80 to 2.15
Anterior basolateral nucleus amygdala	−2.45 to −2.85
Central nucleus amygdala	−2.45 to −2.85
Lateral nucleus amygdala	−2.45 to −2.85
Nucleus accumbens: core and shell	+2.80 to 0.45
Paraventricular nucleus	−1.53 to +2.00
Posterior basolateral nucleus amygdala	−2.45 to −2.85
**EXPERIMENT 2**	
Anterior cingulate	+2.80 to 2.15
Basolateral nucleus amygdala	−2.45 to −2.85
Central nucleus amygdala	−2.45 to −2.85
Dentate gyrus	−2.45 to −2.85
Dorsal raphe	−7.10 to −8.60
Locus coeruleus	−9.60 to −10.10
Nucleus accumbens	+2.80 to 0.45
Paraventricular nucleus	−1.53 to +2.00

### Statistics for Experiment 1

Due to the presence of occasional outliers, the behavioral data were analyzed by one-way analysis of variance (ANOVA) on rank-transformed data, which is equivalent to the Kruskal-Wallis test [66]. When the overall F test of treatment group equality was significant at the 5% level (p<0.05), planned comparisons among treatment groups were made with the LSD (least significant difference) pairwise comparisons method. When the overall F test was not significant at the 5% level, planned comparisons were made with the Bonferroni method [67].

An independent Student's t-test was first applied to the cFos data with regards to the FC and NFC groups to determine if there was an effect of fear conditioning. This was done in order to determine which brain areas were to be further analyzed for data collection and which could be discarded. If a fear conditioning effect was found (p<0.05), all groups were then counted in appropriate brain areas revealed by the t-test and subsequently analyzed by means of a one-way analysis of variance (ANOVA), followed by post-hoc LSD pairwise comparisons. Logged equivalents were used in order to eliminate skew distributions where necessary.

The set of planned comparisons were as follows: FC vs. NFC; FC vs. FC+Ket; NFC vs. Ket; FC+Ket vs. FC+Ket+Cloz; FC+Ket vs. FC+Ket+Halo; and FC+Ket vs. FC+Ket+LY. Statistical analyses were performed with JMP Release 5.1.1 software or SPSS v.12.

### Design of Experiment 2

The rats were divided into 5 groups: sham control (NFC), fear conditioned (FC), FC+ketamine (Ket), FC+clozapine (Cloz) and FC+Ket+Cloz. Clozapine was obtained from Sandoz Pharma AG, Switzerland and ketamine hydrochloride from Sigma, Germany.

### Tissue collection and punching technique

Fifteen minutes after the test session (day 3), rats were anaesthetized with 5% isoflurane and decapitated; brains were quickly removed and frozen in −80°C. Serial 300 µm coronal sections were made with a cryostat microtome (−15°C) and frozen on dry ice. We identified several fear processing regions [37],[68],[16],[50]. Tissue samples were therefore dissected from the ACC, Nacc, PVN, CEA and BLA, dentate gyrus (DG), dorsal raphe (DR) and locus coeruleus (LC; Fig. 5). The Swanson [65] co-ordinates are given in Table 1.

Dissections were made using a needle punch technique on frozen coronal sections. Three different needle diameters were used in accordance with the size of the area to be punched. Larger areas, such as the ACC, Nacc, PVN and DR, were punched with a 16G needle (1.6×40 mm; Sterican, B.Braun, Germany; one punch≈0.23 mm^2^), while the DG, LC (18G: 1.2×38 mm; Sterican, B.Braun, Germany; one punch≈0.19 mm^2^) and the amygdala nuclei (20G: 0.9×40 mm; Sterican, B.Braun, Germany; one punch≈0.08 mm^2^) were punched with smaller diameter needles. Approximately two punches were taken per hemisphere, per animal. Tissue was homogenized in 100 µl (0.1 M) perchloric acid and then the suspension was centrifuged (13,500 rpm) for 10 minutes. The supernatant was stored at −80°C until further analysis.

### Dopamine and glutamate analysis

Analysis of dopamine and its metabolite dopac, was performed by a Shimadzu LC-10 AD high performance liquid chromatograph equipped with a 15-cm reversed phase column (supelcosil 3 µm, C18, 150×4.60 mm, Bester, Amstelveen, The Netherlands) and an electrochemical detector (ESA, Chelmsford, MA, USA) at a potential setting of 300 mV. The mobile phase consisted of 10% methanol, 4.2 g sodium acetate/l, 150 mg octane sulphonic acid/l adjusted to pH 4.10. The injection volume was 20 µl and the flow rate 1 ml/min.

Analysis of glutamate was performed after derivatization with ortho-phtaldehyde by a Shimadzu LC-10 AD high performance liquid chromatograph equipped with a 15-cm reversed phase column (supelcosil 3 µm, C18, 150×4.60 mm, Bester, Amstelveen, The Netherlands) and a fluorescence detector (Waters 470, fluorescence detection, Waters, Milford, Massachusetts, USA) with extinction and emission wavelengths set at 350 nm and 450 nm, respectively. The mobile phase consisted of 26% methanol, 10 g/l disodiumhydrogenphosphate (Na_2_HPO_4_), 150 mg/l EDTA, 2.19 ml/l tetrahydrofuran and adjusted to pH 5.27. The injection volume was 20 µl and the flow rate 1ml/min.

### Statistics for Experiment 2

The overall group effect was assessed via one-way analysis of variance (glutamate, dopamine) or the Kruskall-Wallis test for non-parametric data (dopac, behavior) using SPSS (Version 12). Parametric *vs*. non-parametric tests were chosen on the basis of normal distribution curves. When the overall F test of treatment group equality was significant at the 5% level (p<0.05), planned LSD pairwise comparisons were made among treatment groups (glutamate, dopamine) and Mann-Whitney U test (dopac, behavior) with significance determined at the p<0.05 level.

## Results

### Behavioral data from Experiment 1

**Figure 3:**
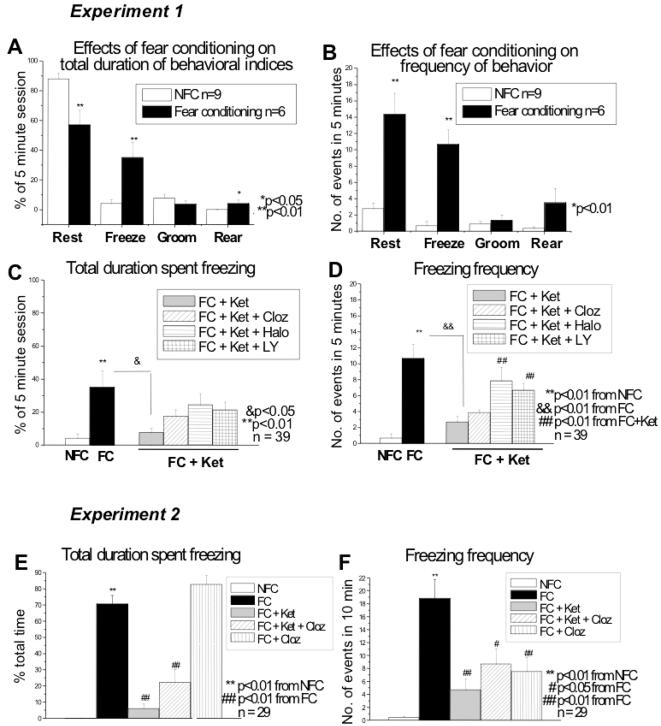
Behavioral data. Experiment 1 (behavior measured after test session): Bars represent means±SEM. Fear conditioning affects almost all of the behaviors including a decrease in resting duration (p = 0.0064; A), and increases in rearing duration (p = 0.0262; A), resting frequency (p<0.0001; B), and freezing duration (p = 0.0001; A) and frequency (p<0.0001; B). As hypothesized, ketamine blocked the effect of fear conditioning (FC vs. FC+Ket), reducing freezing duration (p = 0.0213; C) and frequency (p = 0.0002; d). Haloperidol (p = 0.0040) and LY 379268 (p = 0.0026) were able to partially inhibit this blockade (FC+Ket+Halo/LY vs. FC+Ket), but only in terms of freezing frequency (D). Experiment 2 (behavior measured during test trial): Fear conditioning increased (E) total freezing duration and (f) freezing frequency as compared to the NFC group. Ketamine blocked this effect (FC vs. FC+Ket) in terms of the total duration and freezing frequency. Clozapine alone (FC vs. FC+Cloz) reduced (F) freezing frequency. The FC+Ket+Cloz group was also not statistically different from the FC+Ket group in terms of freezing behavior. *Cloz, clozapine; FC, Fear conditioning; Halo, Haloperidol; Ket, Ketamine; LY, LY 379268; NFC, no fear conditioning.*

The total duration and frequency of behaviors 5 minutes after the test session were analyzed, and are represented in Fig. 3 (Experiment 1). The behaviors of 3 rats in the control group were not included due to technical difficulties with the video recording. The one-way ANOVA revealed significant overall differences for the following behaviors: resting duration (F_6, 38_ = 3.32; p = 0.0099) and frequency (F_6, 38_ = 15.23; p<0.0001), freezing duration (F_6, 38_ = 6.51; p<0.0001) and frequency (F_6, 38_ = 20.42; p<0.0001), and rearing duration (F_6, 38_ = 6.79; p<0.0001) and frequency (F_6, 38_ = 5.35; p = 0.0004).

The LSD pairwise comparisons post hoc showed fear-conditioning effects in most of the behaviors investigated (FC vs. NFC). These include a decrease in resting duration (p = 0.0064; Fig. 3A), and increases in rearing duration (p = 0.0262; Fig. 3A) and resting frequency (p<0.0001; Fig. 3B). More importantly, increases in freezing duration (p = 0.0001; Fig. 3A, C) and frequency (p<0.0001; Fig. 3B, D) were noted.

Ketamine alone did not influence any of the behaviors measured (data not shown). It augmented the effect of fear conditioning with respect to rearing duration (p = 0.0023). In agreement with our hypothesis, however, ketamine blocked the effects of fear conditioning with respect to both freezing duration (p = 0.0213; Fig. 3C) and frequency (p = 0.0002; Fig. 3D).

Comparing the effect of antipsychotics on rats undergoing fear conditioning with ketamine administration (FC+Ket vs. FC+Ket+Cloz/Halo/LY), we find significant differences with respect to rearing duration. Decreases in rearing duration were noted due to clozapine (p = 0.0123) and haloperidol (p = 0.0043) administration, both blocking the effect of ketamine (data not shown). While antipsychotics did not inhibit the effect of ketamine on fear conditioning with respect to freezing duration (Fig. 3C), haloperidol (p = 0.0040) and LY 379268 (p = 0.0026), but not clozapine (p = 0.1033), did block the effect of ketamine with respect to freezing frequency (Fig. 3D).

### cFos data from Experiment 1

**Figure 4:**
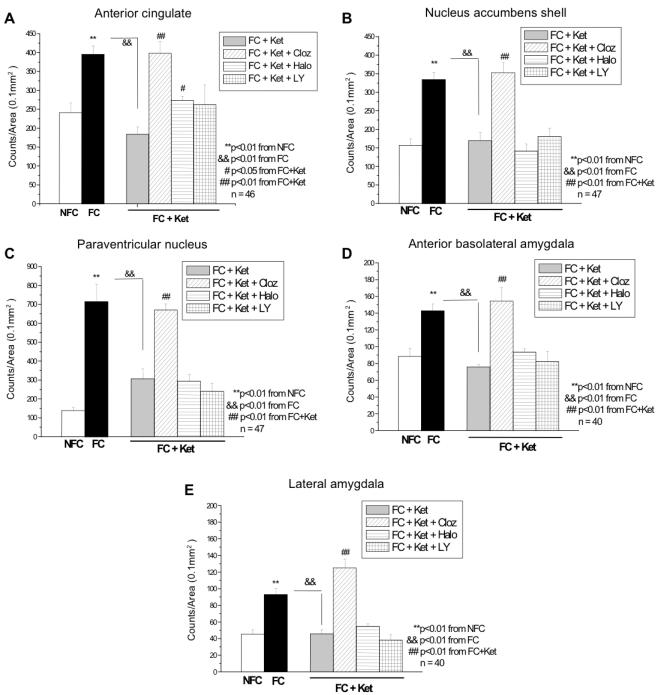
cFos expression. Fear conditioning (FC) increased cFos expression as compared to the NFC group in the anterior cingulate (p = 0.003; A), nucleus accumbens shell (p<0.0001; B), and paraventricular nucleus (p<0.0001; C), anterior basolateral amygdala (p = 0.002; D) and lateral amygdala (p<0.0001; E). Ketamine blocked the effect of fear conditioning (FC vs. FC+Ket) in the anterior cingulate (p<0.0001; A), nucleus accumbens shell (p = 0.002; B), paraventricular nucleus (p<0.0001; C), anterior basolateral amygdala (p = 0.001) and lateral amygdala (p = 0.004). As hypothesized, clozapine was able to counteract the blockade of ketamine on fear conditioning (FC+Ket vs. FC+Ket+Cloz) in the anterior cingulate (p<0.0001), nucleus accumbens shell (p = 0.001), paraventricular nucleus (p = 0.001), anterior basolateral amygdala (p<0.0001) and lateral amygdala (p<0.0001). A slight restoration by haloperidol was noted in the anterior cingulate (p = 0.042). *Cloz, clozapine; FC, fear conditioning; Halo, haloperidol; Ket, Ketamine; LY, LY 379278; NFC, no fear conditioning.*

**Figure 5:**
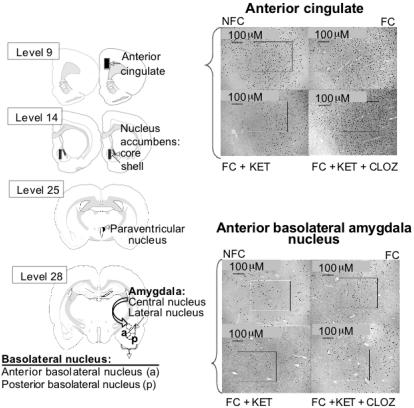
cFos immunocytochemical labeling. Typical examples of the brain areas stained for cFos expression, visually showing the effects of some of the treatments. Delineated areas depict areas measured. Brain slice levels were taken from the Swanson rat brain atlas [65], with appropriate co-ordinates listed in Table 1. *CLOZ, clozapine; FC, Fear conditioning; KET, ketamine; NFC, no fear conditioning.*

Results of the cFos data are represented in Fig. 4, with typical examples of cFos stainings and the delineations of the areas represented in Fig. 5. An independent Student's t-test revealed fear-conditioning effects in the ACC (p = 0.016), Nacc shell (p = 0.001), and the PVN (p<0.0001). No fear conditioning effects were noted in the Nacc core (p = 0.649) and therefore this area was not included for further analyses. With regards to the amygdala, significant fear conditioning effects were found in the anterior portion of the BLA (p = 0.008) and lateral amygdala (p = 0.008), with no effects of fear conditioning in the (medial) central amygdala (p = 0.654) or the posterior portion of the BLA (p = 0.483). The latter two areas were therefore not included in further analyses. The one-way ANOVA revealed significant overall F-tests performed on the remaining groups for the following brain areas: ACC (F_6, 39_ = 5.96; p<0.001), Nacc shell (F_6, 40_ = 8.96; p<0.001), PVN (F_6, 40_ = 25.89; p<0.001), anterior BLA (F_6, 39_ = 9.49; p<0.001) and lateral amygdala (F_6, 39_ = 11.68; p<0.001).

The LSD pairwise comparisons post hoc showed increases in cFos expression due to fear conditioning (NFC vs. FC) in all remaining brain areas: ACC (p = 0.003; Fig. 4A), Nacc shell (p<0.0001; Fig. 4B), and PVN (p<0.0001; Fig. 4C). More importantly, increases due to fear conditioning were noted in the anterior portion of the BLA (p = 0.002; Fig. 4D) and lateral amygdala (p<0.0001; Fig. 4E).

Ketamine led to the hypothesized blocking of cFos expression due to fear conditioning (FC vs. FC+Ket) in all the brain areas investigated, i.e. the ACC (p<0.0001), Nacc shell (p = 0.002), PVN (p<0.0001), anterior BLA (p = 0.001) and lateral amygdala (p = 0.004). Decreases of cFos expression due to ketamine alone (without fear conditioning; data not shown) were also noted in most areas except the ACC (p = 0.087) and Nacc shell (p = 0.09), i.e. PVN (p = 0.047), anterior BLA (p = 0.003) and lateral amygdala (p = 0.01).

It was also hypothesized that clozapine, and not haloperidol or LY 379268 would block the effect of ketamine on fear conditioning (FC+Ket) with regards to cFos expression. These hypotheses were supported by the data, which showed normal fear conditioning effects on cFos expression with clozapine administration. Significant differences between groups due to clozapine administration (FC+Ket vs. FC+Ket+Cloz) were noted in the ACC (p<0.0001), Nacc shell (p = 0.001), PVN (p = 0.001), anterior BLA (p<0.0001) and lateral amygdala (p<0.0001). Haloperidol did show some effect in the ACC, although not as significant as clozapine (p = 0.042). No other effects of haloperidol or LY 379268 drugs were found in any of the other areas investigated.

### Behavioral data from Experiment 2

The Kruskall-Wallis test revealed an overall group effect for the total duration of freezing behavior (χ^2^_4, 24_ = 23.84; p<0.0001; Fig. 3E) and frequency of freezing behavior (χ^2^_4, 24_ = 17.35; p = 0.002; Fig. 3F). Mann-Whitney U post hoc tests showed a significant increase in total duration spent freezing (p = 0.004) and an increase in frequency of freezing (p = 0.004) in the fear conditioned (FC) group relative to the non fear-conditioned group (NFC).

Ketamine significantly blocked the effects of fear conditioning with respect to total duration (p = 0.002) and frequency of freezing behavior (p = 0.004). Clozapine, however, was not able to reinstate fear conditioning levels for either measure of freezing behavior (duration: p = 0.240; frequency: p = 0.310). Clozapine alone decreased freezing frequency (p = 0.009) but not total freezing duration.

### Glutamate data from Experiment 2

Overall group effects were noted in all areas: ACC (F_4, 48_ = 6.46; p<0.0001), Nacc (F_4, 49_ = 9.29; p<0.0001), PVN (F_4, 22_ = 6.29; p = 0.002), CEA (F_4, 43_ = 20.91; p<0.0001), BLA (F_4, 48_ = 8.91; p<0.0001), DG (F_4, 49_ = 18.20; p<0.0001), DR (F_4, 23_ = 94.85; p = 0.006), and LC (F_4, 48_ = 14.45; p<0.0001).

**Figure 6:**
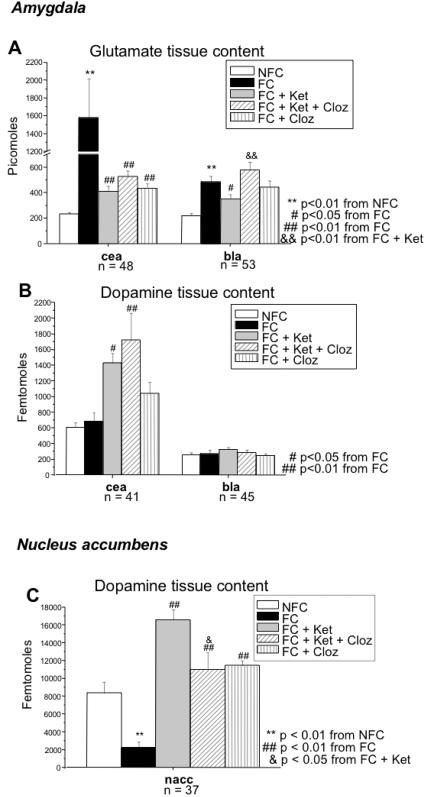
Neurotransmitter content. Bars represent means±SEM. The FC group showed increased glutamate levels (A) in the central and basolateral amygdala, as compared to the NFC group. Ketamine also significantly inhibited this effect in both amygdala nuclei, as revealed by the comparison between FC+Ket and FC groups. Clozapine, in turn, blocked the actions of ketamine on glutamate levels (FC+Ket versus FC+Ket+Cloz) in the central amygdala nucleus, with full restoration of normal fear conditioned-induced glutamate levels in the basolateral amygdala. Clozapine alone (FC+Cloz vs. FC) decreased glutamate levels in the central amygdala. In terms of dopamine content, there were no differences between the FC and NFC groups in either amygdala nuclei (B). A decrease in dopamine content was, however, noted in the nucleus accumbens (C). Ketamine abolished this fear conditioning response, and clozapine partially counteracted the effect of ketamine in the nucleus accumbens. Clozapine alone (no ketamine) also showed effects (FC+Cloz), and increased dopamine content in the nucleus accumbens as compared to the FC group. Ketamine in combination with clozapine (FC+Ket+Cloz; or alone (FC+Ket) increased dopamine levels in the central amygdala (B) as compared to the FC only group. *Bla, basolateral amygdala nucleus; cea, central amygdala nucleus; Cloz, clozapine; FC, fear conditioning; Ket, ketamine; nacc, nucleus accumbens; NFC, no fear conditioning.*

LSD pairwise comparisons (data not shown) revealed increased glutamate content in the FC vs. NFC group in all areas except the ACC (p = 0.099) i.e. Nacc (p<0.0001), PVN (p = 0.012), DG (p<0.0001), DR (p = 0.003), and LC (p<0.0001). Highly significant increases in glutamate were noted in the CEA (p<0.0001) and BLA (p<0.0001; Fig. 6A). Ketamine significantly reduced this effect in these two areas, the CEA (p<0.0001) and the BLA (p = 0.041), in addition to the LC (p = 0.008).

Clozapine prevented the effects of ketamine in the BLA (p = 0.001; Fig. 6A) and LC (p = 0.017). Partial prevention was seen in the CEA (p = 0.088; Fig. 6A). A similar (non-significant) pattern is noted in the PVN and DR. Interestingly, clozapine alone (FC+Cloz), like ketamine, also blocked the increase in tissue glutamate in the central amygdala (p<0.0001; Fig. 6A), and locus coeruleus (p = 0.007; data not shown) associated with fear conditioning.

### Dopamine data from Experiment 2

Overall group analysis shows significant changes of dopamine content in the ACC (F_4, 40_ = 4.18; p = 0.006), Nacc (F_4, 32_ = 15.33; p<0.0001), PVN (F_4, 17_ = 3.15; p = 0.041), CEA (F_4, 36_ = 7.09; p<0.0001), DG (F_4, 17_ = 5.92; p = 0.004), DR (F_4, 18_ = 3.82; p = 0.02) and LC (F_4, 41_ = 5.79; p = 0.001). No significance differences were noted in the BLA in terms of dopamine content (F_4, 40_ = 7.63; p = 0.556).

Fear conditioning induced a decrease in dopamine content in the Nacc (p = 0.005; Fig. 6C) and an increase in the LC (p = 0.006). A trend towards increased dopamine content in the ACC (p = 0.056) is also noted. Ketamine was also able to abolish this fear conditioning (FC+Ket vs. FC groups) response in the Nacc (p<0.0001; Fig. 6C), while showing a trend at augmenting the response of fear conditioning in the ACC (p = 0.053) and LC (p = 0.103). As hypothesized, clozapine partially (b/c FC+K+Cloz in Nacc is still significantly different than FC alone) counteracted the effect of ketamine in these areas: ACC (p = 0.044), Nacc (p = 0.011), and LC (p = 0.008). Ketamine (p = 0.002) increased dopamine content in the CEA (Fig. 6B). Clozapine alone also showed effects (FC+Cloz), increasing dopamine content in the Nacc (p<0.0001; Fig. 6C) and PVN (p = 0.049) as compared to the FC group. Not enough data was available to perform post hoc tests on the DG.

### Dopac/Dopamine ratios from Experiment 2

The Kruskall-Wallis test showed overall group significances in the ACC (χ^2^ (4, 40) = 14.53; p = 0.006), Nacc (χ^2^ (4, 30) = 22.20; p<0.0001) and CEA (χ^2^ (4, 35) = 17.01; p = 0.002). The Mann-Whitney U test revealed an increase in turnover in the Nacc (p = 0.005) due to fear conditioning (Table 2). Ketamine also decreased turnover in the Nacc (p = 0.001) and the CEA (p = 0.008). Clozapine failed to prevent this deficit in both areas (Nacc: p = 0.518; CEA: p = 315).

**Table 2:** The dopac/dopamine metabolic ratios

Brain areas	Control		FC		FC+Cloz		FC+Ket		FC+Ket+Cloz	
	Median	Range	Median	Range	Median	Range	Median	Range	Median	Range
ACC	1.35	2.19	0.79	49.95	0.93	1.79	0.64	0.51	0.82*	2.98
Nacc	0.19	0.16	0.45#	29.08	0.18&	0.04	0.20&	0.05	0.21&	0.14
CEA	0.20	0.17	0.19	13.28	0.20	0.35	0.09&	0.05	0.11+	0.10

* p<0.01 from FC+Ket

# p<0.01 from control

& p<0.01 from FC

+ p = 0.056 from FC

ACC = anterior cingulate, CEA = central amygdala nucleus, Cloz = clozapine, FC = fear conditioned, Ket = ketamine, Nacc = nucleus accumbens

## Discussion

### Summary of results

The main aim of this study was to investigate whether fear conditioning was disrupted in rats following ketamine administration. We hypothesized that ketamine administration induces a hypoglutamatergic state which models negative symptoms (deficits in emotional processing) seen in schizophrenia. Aleman and Kahn [24] proposed that prolonged activation of the amygdala, during psychotic states in the onset stages of schizophrenia, could lead to glutamate excitotoxicity. This would eventually result in amygdala lesions and long-term glutamate hypofunctioning (see also [6]), thereby disrupting a primary brain area in the fear circuit. By administering the NMDA receptor antagonist ketamine, we therefore attempted to simulate disrupted fear processing at a neurochemical level.

We found that fear conditioning alone led to increases in 1) fear-conditioned freezing behavior, 2) cFos expression in the ACC, Nacc shell, PVN, and the anterior BLA and LA nuclei, and 3) increased glutamate tissue content in some brain regions measured, including the amygdala nuclei. Dopamine content was not affected by fear conditioning in most of the brain areas analyzed, with the exception of an increase in the LC and a decrease in the Nacc. In addition, the Nacc showed an increase in dopamine turnover (Table 2).

Ketamine successfully disrupted fear conditioning, both behaviorally and in the measured neural correlates of fear conditioning. Indeed, freezing behavior was decreased almost to control levels. This behavioral abolishment of fear conditioning was also reflected in the ACC, Nacc shell, PVN, anterior BLA and the LA, in terms of reduced cFos expression. Glutamate tissue content was also attenuated down to control (no fear conditioning) levels in the amygdala nuclei, although this was not found to be the case for the ACC. Dopamine content was increased by ketamine administration in the CEA and the Nacc.

We also administered both an atypical and a typical antipsychotic, in addition to a metabotropic glutamate 2/3-receptor (mGlu_2/3_) agonist, LY 379268. As an atypical antipsychotic, clozapine is considered to be useful in ameliorating negative symptoms, whereas the typical antipsychotic, haloperidol, mostly reverses positive symptoms. Other animal studies have indicated that clozapine is successful in blocking metabolic effects induced by ketamine [49], consistent with its action on NMDA receptors. In the same experiment, haloperidol (a preferential dopamine D_2_ receptor antagonist) was not able to block the effects of ketamine. We therefore hypothesized that clozapine would be capable of reversing the effects of ketamine, whereas haloperidol would not. We also tentatively postulated that LY 379268 would prevent ketamine's actions, particularly in forebrain areas.

We found that clozapine administration entirely prevented cFos expression due to subsequent ketamine administration (to fear conditioning levels) in key brain areas regulating fear processing. This included the ACC, Nacc, PVN, anterior BLA and lateral amygdala. With the exception of the ACC, no preventative effects were noted with either haloperidol or LY 379268. Glutamate tissue content levels were also conserved with clozapine administration in the LC and BLA. Dopamine content was also brought closer to fear conditioning levels in the LC and Nacc.

Interestingly, clozapine without ketamine administration (FC+Cloz) had an effect similar to ketamine (FC+Ket), in terms of glutamate content. Glutamate levels induced by fear conditioning were significantly suppressed by clozapine in the central amygdala (Fig. 6A) and locus coeruleus (data not shown), with a trend in the basolateral amygdala (Fig. 6A). These results are consistent with findings showing that clozapine directly suppresses the glutamate system—suggestive of an anxiolytic effect [69],[70]—and that clozapine is the most potent of the antipsychotic agents in blocking NMDA receptor antagonist-induced neurotoxicity [71],[72]. Given the similar neurochemical effects of ketamine and clozapine, and given that ketamine alone had a powerful effect on freezing behavior, it seems puzzling that clozapine did not also have a potent effect on behavior. It is also puzzling to consider why the neurochemical effects of ketamine and clozapine administration do not predict their joint effects when administered together. Since ketamine and clozapine both decrease glutamate levels, we might expect that ketamine and clozapine together should produce even further decreases in glutamate levels. Yet in the basolateral amygdala and the locus coeruleus, ketamine and clozapine administered together led to relatively increased glutamate levels, comparable to the FC group. As clozapine affects several neurotransmitter systems [49],[73],[74], including glutamate, its reversal of ketamine's effect on glutamate could be due to its influence on other neurotransmitters in other brain regions.

Taken together, clozapine appears to block the disruption of fear processing by ketamine in several key brain areas. This effect was not, however, reflected in the behavioral data; we found little evidence to support the idea that clozapine maintains normal freezing behavior following ketamine administration. In order to reconcile the neural and behavioral data, we propose a neurochemically-based conceptual model below.

### Conceptual model

**Figure 7:**
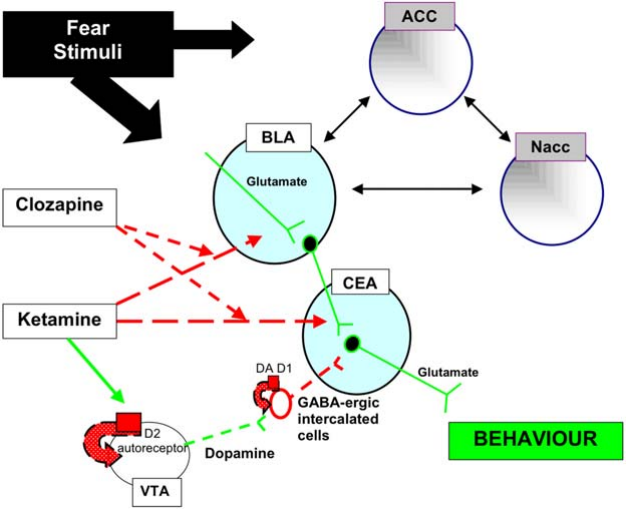
Conceptual model. A schematic drawing of our conceptual model depicting interactions between dopamine and glutamate in the amygdala nuclei. The interactions between other brain areas studied and the amygdala conceptual model are also indicated. Dashed lines symbolize inhibition, while solid lines represent stimulation. Lines between brain areas represent functional connectivity between the regions. Fear stimuli are processed first by the basolateral amygdala (BLA), activating the glutamate system in this area, but do not affect dopamine levels in either the BLA or central amygdala (CEA). Output signals inducing freezing behavior from the CEA are therefore strong via the glutamate pathway. Ketamine decreases glutamate-related fear processing in the BLA and CEA and simultaneously elevates dopamine content (storage) in the CEA, by blocking dopamine release via the dopamine D_2_ autoreceptor located on the cell body (possibly the VTA). The decreased dopamine release (together with the block of glutamate transmission from the BLA to the CEA) blocks the fear-related outputs by means of increased GABA inhibition via intercalated cells projecting onto the CEA. The net effect is weak output signals from the CEA and diminished freezing behavior. Clozapine, while blocking the effects of ketamine on glutamate-related processing in the BLA and CEA, does nothing to prevent changes in dopamine levels. GABA-ergic intercalated cells therefore continue to inhibit CEA and/or BLA and normal fear-conditioned behavior cannot be retained. Can chronic clozapine treatment renormalize dopamine levels and lead to long-term remediation of negative symptoms in the animal model? *ACC, anterior cingulate; DA, dopamine; LC, locus coeruleus; Nacc, nucleus accumbens; PVN, paraventricular nucleus; VTA, ventral tegmental area*

We adapt the model of Aleman and Kahn [24] and Reynolds [75] to the current context in four key ways. First, we propose that glutamate-mediated fear conditioning in the BLA drives freezing behavior through the output nuclei of the CEA [76]–[78],[16]. Second, we hypothesize that dopamine-modulated γ-aminobutyric acid (GABA) inhibition in the CEA modifies the outputs of the BLA [79]. Third, we interpret ketamine administration in terms of a glutamate-mediated deficit in fear conditioning in the BLA [80]–[82],[50]. Lastly, we propose that decreased dopamine turnover in the CEA due to ketamine administration leads to increased GABA inhibition of the BLA's outputs in the CEA, leading to decreased freezing behavior (see Fig. 7). The model can explain the major features of our data as follows.

### Fear conditioning

Glutamate content is increased in both amygdala nuclei as a result of fear conditioning (Fig. 6A), but dopamine content is not affected in either (Fig. 6B). As a result, output signals (glutamate) inducing freezing behavior from the BLA via the CEA remain strong, as dopamine inhibition of GABA interneurons remains intact. In terms of cFos expression, fear conditioning elevates expression in the BLA, but not the CEA. Interestingly, a study by Kleim et al. [83] shows that cFos activity is directly related to learning of a skill, and not only to the execution or maintenance of motor behavior. This could indicate that the BLA is more concerned with processing and storage of fearful memories, while the CEA is mainly an output nucleus [76],[15],[77],[84],[31],[85].

### Ketamine

It has been reported that ketamine administration, in addition to acting on the glutamate system, also acts on the dopamine system, perhaps through direct stimulation of the dopamine D_2_ receptor [86]. The dopamine metabolic ratios in our study (Table 2) indicate that the increase in dopamine content in the CEA after ketamine administration is due to a decrease in turnover (indicative of decreased release and a subsequent increase in storage of the neurotransmitter in axon terminals). That is, less dopamine is acting on the CEA and more is being stored axonally. Ketamine also suppresses glutamate-related fear processing in the BLA and CEA (Fig. 6A), in addition to simultaneously decreasing dopamine turnover in the CEA. We therefore propose that this decreased dopamine release liberates a tonic inhibition by GABA-ergic neurons in the intercalated cells, leading to *increased* inhibition of the glutamate signals from the BLA to the CEA. The net effect is weak output signals from the CEA and consequently diminished freezing behavior.

In agreement with this, Marowsky et al. [87] show that dopamine D_1_ receptor (post-synaptic) activation disinhibits the amygdala by inhibiting GABA-ergic mechanisms within the intercalated cells. Interestingly, systemic application of dopamine D_1_ agonists has been shown to retard or even reverse fear extinction [88], whereas D_1_ antagonists block either the acquisition and/or expression of fear [89],[90]. Decreased dopamine release through ketamine's effects on the dopamine D_2_ autoreceptor in our study may therefore re-activate the inhibitory control of the intercalated cells on the CEA, resulting in behavioral blockade (Fig. 7).

### Antipsychotics

We hypothesized that clozapine but not haloperidol would preserve normal fear-conditioned behavior by blocking the effects of ketamine. We did not find evidence to support this notion. We did, however, find that clozapine prevented the effect of ketamine much more potently than haloperidol and LY 379268 in terms of cFos expression in several brain areas (Fig. 4) and also that it prevents decreased glutamate levels, in the BLA (Fig. 6A) and LC. These observations would appear to predict normal freezing behavior. In terms of the conceptual model, we suggest that normal freezing behavior was not retained because clozapine did not affect the dopamine (and glutamate) levels in the CEA that are observed following ketamine administration. Dopamine turnover and hence pre-synaptic release thereby remained low, leaving the GABA-ergic inhibition of the CEA intact. This unchecked inhibition of the pathway from the BLA to the CEA thus explains why normal freezing behavior was not observed.

### Predictions

What strategies could be used to restore normal fearful behavior? As mentioned previously, ketamine acts primarily as an agonist at the dopamine D_2_ autoreceptor, thereby inhibiting the release of dopamine. We therefore propose two methods to counteract ketamine's effect on fear conditioning: 1) by using a selective dopamine D_2_ antagonist to block ketamine's actions on the dopamine D_2_ autoreceptor or 2) by administering a selective dopamine D_1_ agonist, which directly inhibits GABA-ergic function.

We chose to validate our animal model with clozapine and haloperidol, as they are used in the clinical setting. Because clozapine and haloperidol both act as antagonists at dopamine D_2_ receptors [91], we might suppose that they would block the effect of ketamine on dopamine transmission at this receptor, and preserve normal fear-conditioned behavior. However, both haloperidol and clozapine also have affinities for the dopamine D_1_ receptor [91] and subsequently block the dopamine that might eventually be released as a result of D_2_ autoreceptor blockade, leaving any behavioral blockade via the GABA-ergic cells in place. This could be one explanation why even clozapine is not particularly effective in treating negative symptoms of schizophrenia [92].

We also tentatively postulated the LY 379268 would prevent ketamine's actions, especially in forebrain areas. However, only a small preventative effect was noted in freezing behavior, without any significant changes in cFos expression in forebrain areas. Comparable results were observed in a similar study in our lab [61], where LY 379268 was unable to block ketamine-induced deficits in pre-pulse inhibition. The authors attributed this phenomenon to LY 379268's failure to alter monoamine neurotransmitter content, which could explain the lack of effect on ketamine's inhibition of cFos expression and behavior here.

### Main conclusions regarding original hypotheses

In the Introduction, several hypotheses were outlined with regards to our model of emotional blunting in schizophrenia. Most of the hypotheses were confirmed, except with respect to LY 379268's possible prevention of ketamine effects, and more critically, the potentially restorative effects of clozapine on fearful behavior. We constructed a conceptual model to explain why clozapine did not preserve normal freezing behavior, even though neural correlates of fear conditioning indicated a positive outcome. We also described improvements that may extend the model and allow for full behavioral restoration. Taken together, the current study supports the notion that glutamatergic hypofunctioning in the amygdala and related brain areas underlies deficits in the processing of fear, and could have implications for the mechanisms underlying emotional blunting in schizophrenia. The present study might therefore pave the way for future studies to explore novel drug treatments of these notoriously drug-resistant symptoms, such as selective dopamine D_2_ antagonists or selective D_1_ agonists combined with clozapine.

## References

[1] Peroutka SJ, Synder SH (1980). Relationship of neuroleptic drug effects at brain dopamine, serotonin, alpha-adrenergic, and histamine receptors to clinical potency.. Am J Psychiatry.

[2] Heinz A (2000). [The dopamine hypothesis of schizophrenia. New findings for an old theory] Dopaminhypothese der Schizophrenien. Neue Befunde fur eine alte Theorie.. Nervenarzt.

[3] Jones HM, Pilowsky LS (2002). Dopamine and antipsychotic drug action revisited.. Br J Psychiatry.

[4] Tsai G, Coyle JT (2002). Glutamatergic mechanisms in schizophrenia.. Annu Rev Pharmacol Toxicol.

[5] Coyle JT, Tsai G (2004). The NMDA receptor glycine modulatory site: a therapeutic target for improving cognition and reducing negative symptoms in schizophrenia.. Psychopharmacology (Berl).

[6] Heresco-Levy U (2003). Glutamatergic neurotransmission modulation and the mechanisms of antipsychotic atypicality.. Prog Neuropsychopharmacol Biol Psychiatry.

[7] Hofer E, Doby D, Anderer P, Dantendorfer K (2001). Impaired conditional discrimination learning in schizophrenia.. Schizophr Res.

[8] O'Carroll RE (1995). Associative learning in acutely ill and recovered schizophrenic patients.. Schizophr Res.

[9] Rushe TM, Woodruff PW, Murray RM, Morris RG (1999). Episodic memory and learning in patients with chronic schizophrenia.. Schizophr Res.

[10] Edwards J, Pattison PE, Jackson HJ, Wales RJ (2001). Facial affect and affective prosody recognition in first-episode schizophrenia.. Schizophr Res.

[11] Gur RE, McGrath C, Chan RM, Schroeder L, Turner T (2002). An fMRI study of facial emotion processing in patients with schizophrenia.. Am J Psychiatry.

[12] Johnston PJ, Katsikitis M, Carr VJ (2001). A generalised deficit can account for problems in facial emotion recognition in schizophrenia.. Biol Psychol.

[13] Paradiso S, Andreasen NC, Crespo-Facorro B, O'Leary DS, Watkins GL (2003). Emotions in unmedicated patients with schizophrenia during evaluation with positron emission tomography.. Am J Psychiatry.

[14] Takahashi H, Koeda M, Oda K, Matsuda T, Matsushima E (2004). An fMRI study of differential neural response to affective pictures in schizophrenia.. Neuroimage.

[15] Maren S, Fanselow MS (1996). The amygdala and fear conditioning: has the nut been cracked?. Neuron.

[16] LeDoux J (1998). Fear and the brain: where have we been, and where are we going?. Biol Psychiatry.

[17] Adolphs R, Tranel D, Damasio H, Damasio AR (1995). Fear and the human amygdala.. J Neurosci.

[18] Exner C, Boucsein K, Degner D, Irle E, Weniger G (2004). Impaired emotional learning and reduced amygdala size in schizophrenia: a 3-month follow-up.. Schizophr Res.

[19] Joyal CC, Laakso MP, Tiihonen J, Syvalahti E, Vilkman H (2003). The amygdala and schizophrenia: a volumetric magnetic resonance imaging study in first-episode, neuroleptic-naive patients.. Biol Psychiatry.

[20] Niu L, Matsui M, Zhou SY, Hagino H, Takahashi T (2004). Volume reduction of the amygdala in patients with schizophrenia: a magnetic resonance imaging study.. Psychiatry Res.

[21] Sachdev P, Brodaty H, Cheang D, Cathcart S (2000). Hippocampus and amygdala volumes in elderly schizophrenic patients as assessed by magnetic resonance imaging.. Psychiatry Clin Neurosci.

[22] Taylor SF, Liberzon I, Decker LR, Koeppe RA (2002). A functional anatomic study of emotion in schizophrenia.. Schizophr Res.

[23] Fahim C, Stip E, Mancini-Marie A, Mensour B, Boulay LJ (2005). Brain activity during emotionally negative pictures in schizophrenia with and without flat affect: an fMRI study.. Psychiatry Res.

[24] Aleman A, Kahn RS (2005). Strange feelings: do amygdala abnormalities dysregulate the emotional brain in schizophrenia?. Prog Neurobiol.

[25] Hulshoff Pol HE, Schnack HG, Mandl RC, van Haren NE, Koning H (2001). Focal gray matter density changes in schizophrenia.. Arch Gen Psychiatry.

[26] Castner SA, Goldman-Rakic PS (2003). Amphetamine sensitization of hallucinatory-like behaviors is dependent on prefrontal cortex in nonhuman primates.. Biol Psychiatry.

[27] Grace AA (2000). Gating of information flow within the limbic system and the pathophysiology of schizophrenia.. Brain Res Brain Res Rev.

[28] Marcotte ER, Pearson DM, Srivastava LK (2001). Animal models of schizophrenia: a critical review.. J Psychiatry Neurosci.

[29] Schauz C, Koch M (2000). Blockade of NMDA receptors in the amygdala prevents latent inhibition of fear-conditioning.. Learn Mem.

[30] Schmajuk NA (2001). Hippocampal dysfunction in schizophrenia.. Hippocampus.

[31] Goosens KA, Maren S (2003). Pretraining NMDA receptor blockade in the basolateral complex, but not the central nucleus, of the amygdala prevents savings of conditional fear.. Behav Neurosci.

[32] Yagi K, Onaka T, Yoshida A (1998). Role of NMDA receptors in the emotional memory associated with neuroendocrine responses to conditioned fear stimuli in the rat.. Neurosci Res.

[33] Johnson DM, Baker JD, Azorlosa JL (2000). Acquisition, extinction, and reinstatement of Pavlovian fear conditioning: the roles of the NMDA receptor and nitric oxide.. Brain Res.

[34] Kleim JA, Lussnig E, Schwarz ER, Comery TA, Greenough WT (1996). Synaptogenesis and Fos expression in the motor cortex of the adult rat after motor skill learning.. J Neurosci.

[35] Frankland PW, Bontempi B, Talton LE, Kaczmarek L, Silva AJ (2004). The involvement of the anterior cingulate cortex in remote contextual fear memory.. Science.

[36] Gao YJ, Ren WH, Zhang YQ, Zhao ZQ (2004). Contributions of the anterior cingulate cortex and amygdala to pain- and fear-conditioned place avoidance in rats.. Pain.

[37] Cardinal RN, Parkinson JA, Hall J, Everitt BJ (2002). Emotion and motivation: the role of the amygdala, ventral striatum, and prefrontal cortex.. Neurosci Biobehav Rev.

[38] Han CJ, O'Tuathaigh CM, van Trigt L, Quinn JJ, Fanselow MS (2003). Trace but not delay fear conditioning requires attention and the anterior cingulate cortex.. Proc Natl Acad Sci U S A.

[39] Bush G, Luu P, Posner MI (2000). Cognitive and emotional influences in anterior cingulate cortex.. Trends Cogn Sci.

[40] Reynolds SM, Berridge KC (2003). Glutamate motivational ensembles in nucleus accumbens: rostrocaudal shell gradients of fear and feeding.. Eur J Neurosci.

[41] Salamone JD, Correa M, Mingote SM, Weber SM (2005). Beyond the reward hypothesis: alternative functions of nucleus accumbens dopamine.. Curr Opin Pharmacol.

[42] Johnson LR, Aylward RL, Hussain Z, Totterdell S (1994). Input from the amygdala to the rat nucleus accumbens: its relationship with tyrosine hydroxylase immunoreactivity and identified neurons.. Neuroscience.

[43] Lindefors N, Barati S, O'Connor WT (1997). Differential effects of single and repeated ketamine administration on dopamine, serotonin and GABA transmission in rat medial prefrontal cortex.. Brain Res.

[44] Lorrain DS, Baccei CS, Bristow LJ, Anderson JJ, Varney MA (2003). Effects of ketamine and N-methyl-D-aspartate on glutamate and dopamine release in the rat prefrontal cortex: modulation by a group II selective metabotropic glutamate receptor agonist LY379268.. Neuroscience.

[45] Verma A, Moghaddam B (1996). NMDA receptor antagonists impair prefrontal cortex function as assessed via spatial delayed alternation performance in rats: modulation by dopamine.. J Neurosci.

[46] Levinson DF (1991). Pharmacologic treatment of schizophrenia.. Clin Ther.

[47] Yamamoto BK, Cooperman MA (1994). Differential effects of chronic antipsychotic drug treatment on extracellular glutamate and dopamine concentrations.. J Neurosci.

[48] Daly DA, Moghaddam B (1993). Actions of clozapine and haloperidol on the extracellular levels of excitatory amino acids in the prefrontal cortex and striatum of conscious rats.. Neurosci Lett.

[49] Duncan GE, Leipzig JN, Mailman RB, Lieberman JA (1998). Differential effects of clozapine and haloperidol on ketamine-induced brain metabolic activation.. Brain Res.

[50] Walker DL, Davis M (2002). The role of amygdala glutamate receptors in fear learning, fear-potentiated startle, and extinction.. Pharmacol Biochem Behav.

[51] Swanson CJ, Bures M, Johnson MP, Linden AM, Monn JA (2005). Metabotropic glutamate receptors as novel targets for anxiety and stress disorders.. Nat Rev Drug Discov.

[52] Moghaddam B, Adams BW (1998). Reversal of phencyclidine effects by a group II metabotropic glutamate receptor agonist in rats.. Science.

[53] Cartmell J, Monn JA, Schoepp DD (1999). The metabotropic glutamate 2/3 receptor agonists LY354740 and LY379268 selectively attenuate phencyclidine versus d-amphetamine motor behaviors in rats.. J Pharmacol Exp Ther.

[54] Homayoun H, Jackson ME, Moghaddam B (2005). Activation of metabotropic glutamate 2/3 receptors reverses the effects of NMDA receptor hypofunction on prefrontal cortex unit activity in awake rats.. J Neurophysiol.

[55] Harich S, Gross G, Bespalov A (2007). Stimulation of the metabotropic glutamate 2/3 receptor attenuates social novelty discrimination deficits induced by neonatal phencyclidine treatment.. Psychopharmacology (Berl).

[56] Abekawa T, Ito K, Koyama T (2007). Different effects of a single and repeated administration of clozapine on phencyclidine-induced hyperlocomotion and glutamate releases in the rat medial prefrontal cortex at short- and long-term withdrawal from this antipsychotic.. Naunyn Schmiedebergs Arch Pharmacol.

[57] Bakshi VP, Swerdlow NR, Geyer MA (1994). Clozapine antagonizes phencyclidine-induced deficits in sensorimotor gating of the startle response.. J Pharmacol Exp Ther.

[58] Dunn MJ, Killcross S (2006). Differential attenuation of d-amphetamine-induced disruption of conditional discrimination performance by dopamine and serotonin antagonists.. Psychopharmacology (Berl).

[59] Kapur S, VanderSpek SC, Brownlee BA, Nobrega JN (2003). Antipsychotic dosing in preclinical models is often unrepresentative of the clinical condition: a suggested solution based on in vivo occupancy.. J Pharmacol Exp Ther.

[60] Pietersen CY, Bosker FJ, Postema F, Fokkema DS, Korf J (2006). Ketamine administration disturbs behavioural and distributed neural correlates of fear conditioning in the rat.. Prog Neuropsychopharmacol Biol Psychiatry.

[61] Imre G, Fokkema DS, Boer JA, Ter Horst GJ (2006). Dose-response characteristics of ketamine effect on locomotion, cognitive function and central neuronal activity.. Brain Res Bull.

[62] Bolles RC, Collier AC (1976). The effect of predictive cues on freezing in rats.. Animal Learning and Behavior.

[63] Holahan MR, White NM (2002). Conditioned memory modulation, freezing, and avoidance as measures of amygdala-mediated conditioned fear.. Neurobiol Learn Mem.

[64] Sharp JW (1997). Phencyclidine (PCP) acts at sigma sites to induce c-fos gene expression.. Brain Res.

[65] Swanson LW (1992). Brain maps: Structure of the rat brain..

[66] Montgomery DC (1984). Design and Analysis of Experiments..

[67] Miliken GA, Johnson DE (1992). Analysis of Messy Data Volume 1: Designed Experiments..

[68] Davidson RJ, Irwin W (1999). The functional neuroanatomy of emotion and affective style.. Trends Cogn Sci.

[69] Rex A, Voigt JP, Voits M, Fink H (1998). Pharmacological evaluation of a modified open-field test sensitive to anxiolytic drugs.. Pharmacol Biochem Behav.

[70] Sharma V (2003). Atypical antipsychotics and suicide in mood and anxiety disorders.. Bipolar Disord.

[71] Farber NB, Price MT, Labruyere J, Nemnich J, St Peter H (1993). Antipsychotic drugs block phencyclidine receptor-mediated neurotoxicity.. Biol Psychiatry.

[72] Olney JW, Farber NB (1994). Efficacy of clozapine compared with other antipsychotics in preventing NMDA-antagonist neurotoxicity.. J Clin Psychiatry.

[73] Johnson DE, Nedza FM, Spracklin DK, Ward KM, Schmidt AW (2005). The role of muscarinic receptor antagonism in antipsychotic-induced hippocampal acetylcholine release.. Eur J Pharmacol.

[74] Ma J, Ye N, Cohen BM (2006). Expression of noradrenergic alpha1, serotoninergic 5HT2a and dopaminergic D2 receptors on neurons activated by typical and atypical antipsychotic drugs.. Prog Neuropsychopharmacol Biol Psychiatry.

[75] Reynolds GP (1983). Increased concentrations and lateral asymmetry of amygdala dopamine in schizophrenia.. Nature.

[76] Fanselow MS, Kim JJ (1994). Acquisition of contextual Pavlovian fear conditioning is blocked by application of an NMDA receptor antagonist D,L-2-amino-5-phosphonovaleric acid to the basolateral amygdala.. Behav Neurosci.

[77] Killcross S, Robbins TW, Everitt BJ (1997). Different types of fear-conditioned behaviour mediated by separate nuclei within amygdala.. Nature.

[78] Koo JW, Han JS, Kim JJ (2004). Selective neurotoxic lesions of basolateral and central nuclei of the amygdala produce differential effects on fear conditioning.. J Neurosci.

[79] Pare D, Royer S, Smith Y, Lang EJ (2003). Contextual inhibitory gating of impulse traffic in the intra-amygdaloid network.. Ann N Y Acad Sci.

[80] Miserendino MJ, Sananes CB, Melia KR, Davis M (1990). Blocking of acquisition but not expression of conditioned fear-potentiated startle by NMDA antagonists in the amygdala.. Nature.

[81] Monaghan DT, Cotman CW (1985). Distribution of N-methyl-D-aspartate-sensitive L-[3H]glutamate-binding sites in rat brain.. J Neurosci.

[82] Savonenko A, Werka T, Nikolaev E, Zielinski K, Kaczmarek L (2003). Complex effects of NMDA receptor antagonist APV in the basolateral amygdala on acquisition of two-way avoidance reaction and long-term fear memory.. Learn Mem.

[83] Kleim JA, Lussnig E, Schwarz ER, Comery TA, Greenough WT (1996). Synaptogenesis and Fos expression in the motor cortex of the adult rat after motor skill learning.. J Neurosci.

[84] Shors TJ, Mathew PR (1998). NMDA receptor antagonism in the lateral/basolateral but not central nucleus of the amygdala prevents the induction of facilitated learning in response to stress.. Learn Mem.

[85] Pezze MA, Feldon J (2004). Mesolimbic dopaminergic pathways in fear conditioning.. Prog Neurobiol.

[86] Kapur S, Seeman P (2002). NMDA receptor antagonists ketamine and PCP have direct effects on the dopamine D(2) and serotonin 5-HT(2)receptors-implications for models of schizophrenia.. Mol Psychiatry.

[87] Marowsky A, Yanagawa Y, Obata K, Vogt KE (2005). A specialized subclass of interneurons mediates dopaminergic facilitation of amygdala function.. Neuron.

[88] Borowski TB, Kokkinidis L (1998). The effects of cocaine, amphetamine, and the dopamine D1 receptor agonist SKF 38393 on fear extinction as measured with potentiated startle: implications for psychomotor stimulant psychosis.. Behav Neurosci.

[89] Greba Q, Kokkinidis L (2000). Peripheral and intraamygdalar administration of the dopamine D1 receptor antagonist SCH 23390 blocks fear-potentiated startle but not shock reactivity or the shock sensitization of acoustic startle.. Behav Neurosci.

[90] Inoue T, Izumi T, Maki Y, Muraki I, Koyama T (2000). Effect of the dopamine D(1/5) antagonist SCH 23390 on the acquisition of conditioned fear.. Pharmacol Biochem Behav.

[91] Farde L, Nordstrom AL, Wiesel FA, Pauli S, Halldin C (1992). Positron emission tomographic analysis of central D1 and D2 dopamine receptor occupancy in patients treated with classical neuroleptics and clozapine. Relation to extrapyramidal side effects.. Arch Gen Psychiatry.

[92] Kane JM (1989). The current status of neuroleptic therapy.. J Clin Psychiatry.

